# Medical and pharmacy student concerns about participating on international service-learning trips

**DOI:** 10.1186/s12909-015-0519-7

**Published:** 2015-12-23

**Authors:** Chih Chuang, Siddique H. Khatri, Manpal S. Gill, Naveen Trehan, Silpa Masineni, Vineela Chikkam, Guillaume G. Farah, Amber Khan, Diane L. Levine

**Affiliations:** Department of Medical Education, Wayne State University, 320 East Canfield, Detroit, MI 48201 USA; Department of Internal Medicine, Wayne State University, 4201 St. Antoine, University Health Center 2E, Detroit, MI 48201 USA; Wayne State University, Detroit, MI 48201 USA

**Keywords:** Student concerns, Service learning trips, Global health trips, Short-term medical service trips, Medical missions, Medical volunteerism, Medical students, Pharmacy students

## Abstract

**Background:**

International Service Learning Trips (ISLT) provide health professional students the opportunity to provide healthcare, under the direction of trained faculty, to underserved populations in developing countries. Despite recent increases in international service learning trips, there is scant literature addressing concerns students have prior to attending such trips. This study focuses on identifying concerns before and after attending an ISLT and their impact on students.

**Methods:**

A survey comprised of closed and open-ended questions was developed to elucidate student concerns prior to attending an ISLT and experiences which might influence concerns. A five-point Likert-scale (extremely concerned = 1, minimally concerned = 5) was used to rate apprehension and satisfaction. Paired *t*-test was used to compare pre- and post-trip concerns; Chi-Square test was used to compare groups.

**Results:**

Thirty-five students (27 medical, 8 pharmacy) attended ISLTs in December 2013. All completed pre and post-trip surveys. Significant decreases were seen in concerns related to cultural barriers (4.14 vs 4.46, *P* = .047), disease/epidemics (3.34 vs 4.60, *P* < .001), natural disasters (3.94 vs 4.94, *P* < .001), terrorism (4.34 vs 4.94, *P* < .001), travel (3.86 vs 4.51, *P* < .001) monetary issues (3.80 vs 4.60, *P* < .001), hospitality (3.94 vs 4.74, *P* = .001) and food (3.83 vs 4.60, *P* < .001). Language and group dynamics remained concerns post-trip. On open-ended questions, students described benefits of attending an ISLT.

**Conclusions:**

Students had multiple concerns prior to attending an ISLT. Most decreased upon return. Addressing concerns has the potential to decrease student apprehension. The results of this study highlight the benefits of providing ISLTs and supporting development of a curriculum incorporating trip-related concerns.

**Electronic supplementary material:**

The online version of this article (doi:10.1186/s12909-015-0519-7) contains supplementary material, which is available to authorized users.

## Background

International medical service learning trips (ISLT), also known as global health trips or medical missions, encompass trips of varied durations that provide opportunities for health professional students to deliver medical care and health education, under the direction of trained health providers, to underserved communities in low income and developing countries. There is extensive interest in ISLT by North American medical students. Participation by US medical students increased from 6 % in 1984 to nearly 20 % in 2003. By 2011, nearly two thirds of U.S. medical students planned to participate in some form of global health learning or services during medical school [1].

As the prevalence of international service-learning trips (ISLTs) increases, there is growing interest in the impact such trips have on medical students [2, 3]. It is commonly asserted that they improve communication, listening, and clinical diagnosis skills of students by putting them in a setting where human interaction takes precedence over technology [4]. Students acquire firsthand knowledge of common diseases in the developing world and become acquainted with public health issues in resource-poor settings [4–6]. Furthermore, relief trips foster clinical competence and cultural awareness among students by giving them the opportunity to provide medical care in underserved regions [5, 7, 8]. Additionally, medical students who have participated in such trips tend to have higher National Board of Medical Examiners Part II scores compared to those who have not participated [9]. Therefore, global health opportunities not only sharpen the clinical acumen of students but also give them an edge by exposing them to a side of medicine that cannot be experienced in the classroom.

Despite recent increases in global health trips, there is scant literature addressing specific concerns students have prior to attending such trips [2, 3]. Our study focuses on identifying student concerns before and after attending a service-learning trip and the impact on student satisfaction and achievement of personal and professional goals.

## Methods

We conducted a literature review to identify articles related to students’ participation in global health missions with a focus on student concerns. We utilized PubMed and Google Scholar to conduct our search and used the following terms: “Global health missions”, “medical missions”, “international service learning trips (ISLTs)”, “short-term ISLTs”, “student experiences on global health trips”, “student concerns and global health trips.” Variables of interest including monetary issues, travel issues, terrorism, disease/epidemics, language, and cultural barriers were derived from the literature [4, 10–12]. Questions relating to these themes were developed. We asked faculty and students who had attended multiple ISLTs in the past to provide input regarding concerns they personally experienced or observed in others. We modified and added questions. To enhance content validity we asked these same individuals to review the final surveys to ensure we identified all relevant concerns. After refining questions, we piloted pre and post trip surveys to a sample of ten students who had not attended a previous ISLT. We identified redundancies and shortened the survey to enhance usability.

The final surveys included questions on demographics, foreign language skills, travel history, history of attending previous global health trips, and questions related to financing the trip. In the pre-trip survey, we collected data on levels of apprehension related to language, food, hospitality, diseases and epidemics, natural disasters, terrorism, travel concerns, monetary concerns, cultural barriers, religious barriers and group dynamics. Additional information was obtained by asking the participants about the topics researched before the trip and resources used to prepare for the trip. Following the trip, students completed a post-trip survey to assess their concerns, guidance they received from senior students and faculty, and overall satisfaction with the experience. We used a five-point Likert-type scale (1-extremely concerned; 5-minimally concerned) to rate the level of apprehension related to concerns both before and after the trip and the level of guidance students received from senior students and faculty (1-extremely helpful; 5 minimally helpful). We also used a Likert-scale to determine the likelihood of students participating in similar trips in the future (1-very likely; 5-never). Dichotomized (yes, no, undecided) questions were used to determine if students would recommend the trip to their peers, if they still wanted to practice medicine overseas at any point in their career and whether such trips should be integrated into their school’s curriculum. An exploratory question to determine the impact of an ISLT on political engagement was included to provide baseline information for future research. We specifically asked students if they had voted in the previous presidential election and if so, whether their awareness of global health issues influenced their vote.

Previous studies have described the effects of short-term ISLTs on students but have not addressed the influence of previous life experiences [2, 4, 6]. We asked a number of open-ended questions to gain a deeper understanding of how these experiences might influence students’ concerns. We were specifically interested in gaining insight on the impact of having lived or traveled abroad, ability to speak the native language, previous exposure to health-care outside the United States, and why they chose to go to a particular country. We also assessed students’ views regarding the personal and professional benefits of participation.

After the trip, students identified reference tools they took with them and any barriers that prevented them from utilizing these tools. They also described what they learned in terms of clinical skills. Students described how their experience affected their view of global health and how it influenced plans to participate in global health after graduation. Lastly, students were asked to provide feedback on how the survey and the trip itself could be improved in the future. The pre-trip survey contained 38 questions (see Additional file 1). The post-trip survey contained 36 questions; demographic questions were not repeated (see Additional file 1). Our study was approved by the Human Investigation Committee at Wayne State University (IRB # 129712B3X). Participation on the ISLT was not contingent on participation in the study.

We administered the surveys to medical and pharmacy student members of the World Health Student Organization (WHSO) at the Wayne State University School of Medicine (WSU-SOM) who were participating on a short term ISLT in December 2013.

We administered the pre-trip survey to students at an informational meeting two weeks prior to the trip. Each student chose a unique code (a color and six digit number), which was marked on each of their surveys to keep their identities anonymous and allow for paired analysis of pre-trip and post-trip data. The post-trip survey was completed at a debriefing session within two weeks of their return. We used bivariate analyses with paired *t*-test to compare the pre-trip and post-trip survey data along with qualitative analysis on our open-ended questions. The Chi-Square test was used to analyze a portion of the pre-trip and post-trip data. *P* value of 0.05 was considered significant. We used IBM SPSS Statistics Version 21 (IBM, Armonk, NY, USA).

Three members of the research team reviewed responses from open-ended questions. A master list of responses was created. Inductive codes were formulated to identify and pool similar concepts [13]. Responses were again studied to check the appropriateness of the codes applied to the data [13]. Codes were then grouped by their nature into themes. Codes were reconciled by consensus and themes were identified [13].

## Results

All students provided informed consent and returned the survey. For questions which were not answered, we calculated percentages based on the number of students who responded to the question. *P* values for appropriate questions were calculated based upon the total number of students who answered the question. Demographic data is provided in Table 1. Out of thirty-five total students, thirty (86 %) were born in the United States [14]. All students spoke English as their first language and had graduated from an undergraduate institution in the United States [14]. Seven out of thirty students (23 %) indicated they had lived outside the United States for at least one year; none had lived in a third world country [14]. Many participants specified they spoke another language in addition to English, with Spanish being the most common [14]. Out of 35, eleven (31 %) students spoke Spanish at a conversational level and one student (3 %) spoke fluently [14].

**Table 1:** Student demographics

Demographics	Total (*N* = 35)
Male	46 %
Female	54 %
White	74 %
Black	6 %
Asian	14 %
Other	6 %
Medical School students	77 %
Pharmacy School students	23 %

Forty percent (14/35) of the participants had previously attended a WHSO trip. Students gave a variety of reasons for selecting this trip as their destination [14]. These included prior visits to the country, the desire to experience a new culture and to help the local population, the desire to learn a new language, and the total cost of the trip [14].

Students shared their personal and professional goals on the pre-trip survey. In terms of personal goals, students commented that they hoped to learn about new cultures and health systems and build relationships with other students [14]. In terms of professional goals, students expressed desires to have an opportunity to help people and improve their clinical skills and medical knowledge [14].

**Fig. 1:**
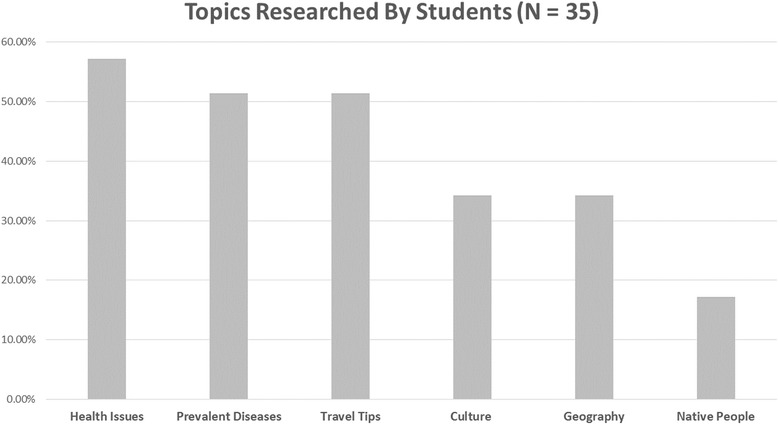
Topics researched by students prior to trip

Students utilized a variety of resources to research information about their destination country before the trip. Web-based resources including Google, Centers for Disease Control and Prevention, and Wikipedia® were utilized by 29 of 35 students (83 %) [14]. Six students (17 %) reported using a smart phone/electronic device application, and four students (11 %) used a travel book/guide [14]. There were a variety of topics researched (see Fig. 1). Students researched the diseases prevalent in the area (18/35; 51 %), languages spoken (18/35; 51 %), and travel tips (18/35; 51 %), while geography (12/35; 34 %), culture (12/35; 34 %) and information about native peoples (6/35; 17 %) were reviewed less often [14].

Students had various concerns prior to the trip (see Table 2). They were most concerned about disease and epidemics and least concerned about religious barriers [14]. All concerns, except for those related to language, decreased after the trip. Analysis of pre-trip and post-trip Likert responses (1-extremely concerned; 5-minimally concerned) using paired *t*-test demonstrated a significant decrease in concerns related to cultural barriers, diseases and epidemics, natural disasters, terrorism, travel and monetary issues, hospitality, and cuisine (see Table 2). Concerns related to religious barriers and group dynamics did not significantly decrease but there were low levels of concern in these domains both before and after the trip. Language was the only concern which increased after the trip. In contrast, students who spoke Spanish displayed a near-significant decrease in their concerns related to language following the trip compared to those who did not speak Spanish (*P* = .053). Students who researched prevalent diseases in the destination country had significantly less pre-trip concerns related to disease than those who did not research this aspect (*P* = .02). Previous participation on service-learning trips had no impact on student pre-trip concerns. There were no differences in pre-trip or post trip levels of concerns in any domain by gender, previous attendance on WHSO trip, experience living abroad or whether the student was a pharmacy or medical student. Finally, student concerns had no impact on the likelihood of incorporating global practice into their future career plans.

**Table 2:** Average Likert score for pre and post-trip students’ concerns

Student Concern	Pre-trip^a^	Post-trip^b^	*P*-value^c^
Disease/Epidemics	3.34	4.60	< .001
Language	3.74	3.37	.079
Monetary Issues	3.80	4.60	< .001
Food	3.83	4.60	< .001
Travel	3.86	4.51	< .001
Group Dynamics	3.91	4.37	.081
Natural Disasters	3.94	4.94	< .001
Hospitality	3.94	4.74	.001
Cultural Barriers	4.14	4.46	.047
Terrorism	4.34	4.94	< .001
Religious Barriers	4.43	4.71	.096

^a.^ Lower values indicate higher levels of concern

^b.^ Likert scale 1–5 with 1 – Extreme Concern and 5 – Minimal Concern

^c.^ *P* value ≤ .05 indicates statistical significance

In order to communicate better with their patients, students used various language aids. A majority (29/34; 85 %) relied on local volunteer medical translators [14]. Twelve students (35 %) used a smartphone or electronic device, eight (24 %) used a bilingual dictionary/thesaurus, and seven (21 %) used a language-learning program [14]. Students utilized a variety of resources to finance their trip [14]. A majority (20/34, 59 %) used student loans to cover the costs. Sixteen (46 %) received assistance from family members, ten (29 %) fund-raised, and five (14 %) used other means to pay for their trip [14].

Students were involved in a group sustainability project before and during the trip involving the distribution of water filters to the local population. Twenty-two out of thirty-five students (63 %) indicated they felt their projects were worthwhile [14].

Thirty-four of thirty-five students (97 %) stated they were satisfied with their trip experience, thirty-two (91 %) stated they were likely to participate in a future trip as a student, and thirty-three (94 %) indicated that they would likely participate in future trips as faculty members [14]. Finally, as a consequence of the trip, more students planned to incorporate global practice into their future career plans (pre-trip: 20/34, 59 %, post-trip: 25/34, 74 %; *P* < .001).

According to US census data, 21 % of the population aged 18–29 was eligible to vote in the 2012 presidential election and 15 % of total votes cast in the election were from this age group [15]. Furthermore, their voting rate in the election was 45 % [15]. Sixty-five percent (23/35) of students who attended this ISLT indicated they had voted in the most recent presidential election, while 25 % (9/35) did not vote [14]. Eight percent (3/35) chose the “prefer not to answer” option on this question [14]. A majority (16/28; 57 %) of students indicated global health issues did not influence their vote while 28 % (8/28) indicated it did have an impact on their decision to vote [14]. Finally, 14 % (4/28) of students chose the “prefer not to answer” option to this question [14].

Analysis of comments from an open-ended question asking about personal and professional goals demonstrated student’s pre-trip expectations were met and, in most cases, surpassed. On a personal level, many expressed an enhanced level of cultural awareness, exposure to a different health-care environment, and an increase in passion for helping the underserved. On a professional level, many students noticed an improvement in their clinical skills, enjoyed the opportunity to work with physicians and senior students, and enhanced language skills, all while caring for patients (see Table 3).

**Table 3:** Themes identified from student comments

Theme(s)	Representative students’ comments
Empathy	➢ “A great ‘life’ learning experience. A trip that solidifies compassion to a new degree.”
Cultural Awareness	➢ “Learned new culture – Friendship”
	➢ “Exposure to providing health-care to a group of people with a different culture and language”
Professional Development	➢ “Did my first surgery, saw my first patient, networked with doctors and other students, and got inspired for using my skills in my own under-served areas and those abroad.”
	➢ “I learned a lot of clinical skills, including physical exam techniques and perhaps more importantly communication skills.”
Impact on patient population/Impact on health professional	➢ “Exposure to health-care in a completely different country and patient population.”
	➢ “Belief that we have a positive impact on people’s lives.”

## Discussion and conclusions

Students identified multiple concerns prior to participation on an international service-learning trip. Concerns included disease and epidemics. All concerns, except for those related to language, decreased after the trip. Nearly all students provided positive feedback in their surveys and reported being extremely satisfied with the overall experience. These students were influenced on both a personal and professional level. Post-trip, students reported the trip provided them with exposure to clinical medicine, an opportunity to enhance their patient interviewing and physical diagnosis skills, a greater awareness of global health issues, and the opportunity to work in a resource-poor setting. Most students expressed a desire to attend additional medical relief trips and to encourage other students to do so as well.

Prior studies, which reported on similar student experiences, have concluded that exposure to health-care in an underserved region should be offered during medical school [8]. It was also concluded that these international experiences enhance self-awareness of students and aid in the development of their world-view [8]. This may explain why the American Association of Medical Colleges (AAMC) is undertaking an expansion in their Global Health Learning Opportunities (GHLO) program [2, 9, 16]. Currently, twelve US medical schools participate in the GHLO program, including WSU-SOM [16]. The program’s primary purpose is to facilitate international exposure of medical students [16]. Along with their objective of encouraging students to acquire international experiences, they aim to provide students with the guidance and resources necessary to address any pre-trip concerns they may have.

Identifying students’ pre-and-post trip concerns provides insight into how to best prepare students for global health learning opportunities. Addressing concerns prior to the trip by developing a pre-trip curriculum and incorporating education about prevalence of disease and epidemics, cultural and religious barriers, issues related to money, food, hospitality and travel, education on group dynamics and team functioning have the potential to further optimize student experiences. Curricula can be developed for specific trips, regions and students [10]. The AAMC’s GHLO program can create an opportunity for participating medical schools to contribute resources for use at other medical schools.

Selection of students with specific language skills has the potential to decrease student language concerns. Alternatively, offering courses in basic medical Spanish (or other appropriate languages) may help better address this particular concern, decreasing pre-trip anxiety and thereby providing a better learning experience. Future studies should analyze language barriers that persist despite the preliminary preparations undertaken by students.

The results of this study further highlights the need for not only providing international experiences to students, but also for the development of a global health curriculum to address important issues related to travel which cause students concern. According to the AAMC, the annual median tuition rates of medical schools in the United States are $34,540 and $53,714 for public and private schools, respectively [17]. The average debt of a medical student after graduation is approximately $176,348 [17]. Therefore, it is worth noting a majority of students in our study financed their trip using student loans. Considering the average cost per student to cover travel was $1800, students were truly motivated to attend the global health trips despite the potential increase in overall debt burden.

There are limitations to this study. Although the post-trip survey was completed within two weeks of the trip, results were subject to recall bias. Additionally, this is a small study of thirty-five students at a single U.S. institution. Our results may not be applicable to other medical schools. Nevertheless, our study highlights the importance of identifying students’ trip-related concerns. Finally, addressing such concerns, through implementation of a global health curriculum, will further enhance the value of ISLTs.

Despite concerns identified by students prior to attending a service-learning trip, most concerns decreased upon their return and their satisfaction with the trip experience was clearly expressed. Addressing concerns prior to the trip has the potential to decrease pre-trip anxiety and further enhance these experiences. Future studies should address the impact of trip-specific curricula to optimize global health learning experiences.

## Additional file

Additional file 1:
**Pre-Trip Survey.** (DOCX 21 kb)
