# Supplementary material for: Medical and pharmacy student concerns about participating on international service-learning trips
Source: BMC Med Educ. 2015 Dec 23;15:232. doi: 10.1186/s12909-015-0519-7 (PMC4690328; doi:10.1186/s12909-015-0519-7)
Supplement: Additional file 1: — Pre-Trip Survey. (DOCX 21 kb) [file 12909_2015_519_MOESM1_ESM.docx]

**Appendix**

**Pre-Trip Survey**

The following survey is part of a pilot study on medical relief trips and their impact on the personal and professional lives of the individuals who attend them. In following survey, please answer every question as fully as possible and write any additional information or comments that might help us better understand your responses. All answers are confidential and will be kept anonymous.

Age: ____

Sex:

□ Male

□ Female

Ethnicity:

□ Hispanic or Latino

□ Not Hispanic or Latino

Race:

□ White or European American

□ Black or African American

□ Asian

□ American Indian or Alaska Native

□ Other race or two/more races (please specify) ______________________________________________

Place of Birth:

City: ________________ State/Province: ________________ Country: ________________

Please list all places you have lived (for at least 1 year) outside of the United States

City: __________ Country: __________ # of Year(s): __________

City: __________ Country: __________ # of Year(s): __________

City: __________ Country: __________ # of Year(s): __________

City: __________ Country: __________ # of Year(s): __________

City: __________ Country: __________ # of Year(s): __________

Please indicate your first language. ______________________________

List any other languages you speak. Indicate whether you are fluent or conversational

(circle one).

1. Language: ________________ Fluent Conversational
2. Language: ________________ Fluent Conversational
3. Language: ________________ Fluent Conversational

What was the average household income per year in the house you grew up in?

□ Less than $50,000

□ $50,000-$75,000

□ $75,000-$100,000

□$100,000-$200,000

□ More than $200,000

How are you currently paying for school? Please select all that apply.

□ Assistance from family

□ Student Loans

□ Personal Savings

□ Other (please specify): ________________________________________________________________

How did you go about paying for this trip? Select all that apply.

□ Assistance from family

□ Student Loans

□ Fundraising

□ Personal Savings

□ Other (please specify): ________________________________________________________________

Undergraduate Institution: _______________________________________________________________

Major: ___________________________________________

Degree received: BS BA

Have you earned a post-graduate degree prior to entering your current post-graduate institution? (i.e: Master’s degree, PhD)

□ Yes

Degree: ___________________________________________________

Institution: _________________________________________________

□ No

Current Post-graduate institution: _________________________________________________________

Type of school

□ Medical School

□ Pharmacy School

□ Nursing School

□ Dentistry School

□ Other (please specify) ____________________________

Year of current schooling (circle one):

Undergraduate 1 2 3 4

Have you attended any medical missions outside of the WHSO? If yes, please indicate how many and with which organization.

□ No

□ Yes

__________________________________________________________________________________________________________________________________________________________________________________________________________________________________________

How many WHSO trips have you attended? 0 1 2 3 4 5+

Please specify where and when you traveled for your WHSO trips.

1. Country: _________________________ Date (mm/yyyy):______________________
2. Country: _________________________ Date (mm/yyyy):______________________

Please select which country you will be traveling to for this upcoming medical relief trip

□ Ecuador □ Haiti □ Peru □ Nicaragua □ Panama

What influenced your decision to travel to the country you selected in the previous question? Please select all that apply.

□ Expense of trip

□ Humanitarian reasons

□ Want to experience a new culture/environment

□ Mission trip work related to desired specialty

□ Prior personal experience

□ Other students' experience

□ Other (please specify): ______________________________________________________

Describe any exposure you have had to medicine and/or healthcare outside of the United States.

______________________________________________________________________________________________________________________________________________________________________________________________________________________________________________________________________________________________________________________________________________________________________________________________________

Have you ever provided healthcare outside the US/Canada?

□Yes

□ No

Have you ever been a patient in a healthcare system outside of the US/Canada?

□Yes

□ No

Describe any experiences you have had living or traveling abroad.

______________________________________________________________________________________________________________________________________________________________________________________________________________________________________________________________________________________________________________________________________________________________________________________________________

Please list the specialties you are considering at this time in order of preference (One specialty per line). Also, please rate your level of commitment to each specialty. (1 -- Minimal; 2 -- Slight; 3 -- Moderate; 4 -- Considerable; 5 -- Maximal)

1. _______________________________________ 1 2 3 4 5
2. _______________________________________ 1 2 3 4 5
3. _______________________________________ 1 2 3 4 5
4. _______________________________________ 1 2 3 4 5
5. _______________________________________ 1 2 3 4 5

Does the specialty of the faculty members attending this trip have any influence in your choice to attend this particular trip?

□ Yes

□ No

□ Unaware of specialties of faculty members

Are you considering practicing medicine overseas at any point in your career?

□ Yes

□ No

Do you plan to practice medicine overseas 1-2 weeks at a time at any point in your career?

□Yes

□ No

Do you plan to practice medicine overseas for a period of greater than 1 year at a time at any point in your career?

□ Yes

□ No

How likely are you to participate in another medical relief trip after this upcoming one?

□ Very likely

□ Likely

□ Neutral/Not sure

□ Not likely

□ Never

What are you hoping to gain from this trip on a personal level?

______________________________________________________________________________________________________________________________________________________________________________________________________________________________________________________________________________________________________________________________________________________________________________________________________

What are you hoping to gain from this trip on a professional level?

______________________________________________________________________________________________________________________________________________________________________________________________________________________________________________________________________________________________________________________________________________________________________________________________________

Rate your level of apprehension with each of the following aspects of the trip.

Extreme Considerable Moderate Slight Minimal

Language □ □ □ □ □

Food □ □ □ □ □

Hospitality □ □ □ □ □

Disease/health epidemics □ □ □ □ □

Natural disasters □ □ □ □ □

Terrorism □ □ □ □ □

Travel concerns □ □ □ □ □

Monetary issues □ □ □ □ □

Cultural barriers □ □ □ □ □

Religious barriers □ □ □ □ □

Group dynamics □ □ □ □ □

Other (please specify):____________________________________________________________

Which topics did you research in preparation for your trip? Select all that apply.

□ Prevalent diseases in destination country

□ Health issues in the country

□ Geography

□ Culture (food, traditions, norms, etc.)

□ Native people

□ Language

□ Travel tips

□ Other (please specify): ________________________________________________________________

□ I did not do any research

Which resources did you use for your research? Select all that apply.

□ Search engines/other online sources (Google, Wikipedia, CDC website)

□ Encyclopedia

□ Textbook

□ Travel book/guide

□ Smartphone/electronic device applications

□ Spoke to people who have traveled to the destination country

□ Other (please specify):

­­­­­­­­­­­­­­­­­­­­­­­­­­­­­­________________________________________________________________________

□ I did not do any research

How are you planning on facing the language barrier during your trip? Select all that apply.

□ Language learning program prior to trip (Rosetta Stone, Pimsleur, etc.)

□ Assistance from a human translator during trip

□ Smartphone/electronic device application (Google Translator, Bing Translator, etc.)

□ Bilingual dictionary, thesaurus, or similar reference tool

□ Other (please specify): ______________________________________________________________________________

Please indicate any other reference tools you are taking on your trip. Select all that apply.

□ Language/communication aids

□ Diagnostic aids

□ History/physical examination aids

□ Other (please specify):_______________________________________________________________________

Please include any suggestions you have which could help us improve the workshop for future students.

__________________________________________________________________________________________________________________________________________________________________________________________________________________________________________________________________________________________________________________________________________________________________________________________________________________________________________________________________________________________________________________________________________________________________

Please include any changes we could make to improve the overall quality of this survey.

__________________________________________________________________________________________________________________________________________________________________________________________________________________________________________________________________________________________________________________________________________________________________________________________________________________________________________________________________________________________________________________________________________________________________

**Post-Trip Survey**

The following survey is part of a study on medical relief trips and their impact on the personal and professional lives of the individuals who attend them. In following survey, please answer every question as fully as possible and write any additional information or comments that might help us better understand your responses. All answers are confidential and will be kept anonymous.

Rate your overall level of satisfaction with the trip.

□ Extreme

□ Considerable

□ Moderate

□ Slight

□ Minimal

How likely are you to participate in another medical relief trip as a student?

□ Very likely

□ Likely

□ Neutral/Not sure

□ Not likely

□ Never

How likely are you to participate in another medical relief trip, in the future, as a faculty member?

□ Very likely

□ Likely

□ Neutral/Not sure

□ Not likely

□ Never

What did you gain from this trip on a personal level?

______________________________________________________________________________________________________________________________________________________________________________________________________________________________________________________________________________________________________________________________________________________________________________________________________

What did you gain from this trip on a professional level?

______________________________________________________________________________________________________________________________________________________________________________________________________________________________________________________________________________________________________________________________________________________________________________________________________

Would you recommend this medical relief trip to other medical students?

□ Yes

□ No

Are you considering practicing medicine overseas at any point in your career?

□ Yes

□ No

Do you plan to practice medicine overseas 1- 2 weeks at a time at any point in your career?

□Yes

□ No

Do you plan to practice medicine overseas for a period of greater than 1 year at a time at any point in your career?

□ Yes

□ No

Do you feel that a short-term international health experience should be integrated into the medical/pharmacy school curriculum as a requirement?

□ Yes

□ No

Do you feel that a clinical elective in an underserved area in your local community should be integrated into the medical/pharmacy school curriculum as a requirement?

□ Yes

□ No

What are the specialties of the faculty members who attended the trip?

1. Primary specialty: __________________________________
2. Primary specialty: __________________________________
3. Primary specialty: __________________________________
4. Primary specialty: __________________________________

What are the prospective specialties of the senior students who attended the trip?

1. Primary specialty: __________________________________
2. Primary specialty: __________________________________
3. Primary specialty: __________________________________
4. Primary specialty: __________________________________

Please list the specialties you are considering at this time in order of preference (One specialty per line). Also, please rate your level of commitment to each specialty. (1 -- Minimal; 2 -- Slight; 3 -- Moderate; 4-- Considerable; 5 -- Maximal)

1. _______________________________________ 1 2 3 4 5
2. _______________________________________ 1 2 3 4 5
3. _______________________________________ 1 2 3 4 5
4. _______________________________________ 1 2 3 4 5
5. _______________________________________ 1 2 3 4 5

Did preference for specialties change after the trip? If so, explain why.

______________________________________________________________________________________________________________________________________________________________________________________________________________________________________________________________________________________________________________________________________________________________________________________________________

Did your level of commitment to each specialty change after the trip? If so, explain why.

________________________________________________________________________________________________________________________________________________________________________________________________________________________________________________________________________________________________________________________

Did the specialty of the faculty members influence your preference in specialty?

□ Yes

□ No

□ Undecided

Rate the level of helpfulness of the senior students in addressing any questions/concerns.

□ Extreme

□ Considerable

□ Moderate

□ Slight

□ Minimal

Rate the level of helpfulness of the faculty in addressing any questions/concerns.

□ Extreme

□ Considerable

□ Moderate

□ Slight

□ Minimal

Rate the level of trouble you had with each of the following aspects of the trip.

Slight Minimal Extreme Considerable Moderate

Food □ □ □ □ □

Language □ □ □ □ □

Hospitality □ □ □ □ □

Disease/health epidemics □ □ □ □ □

Natural disasters □ □ □ □ □

Terrorism □ □ □ □ □

Travel concerns □ □ □ □ □

Monetary issues □ □ □ □ □

Cultural barriers □ □ □ □ □

Religious barriers □ □ □ □ □

Group dynamics □ □ □ □ □

Other (please specify):____________________________________________________________

On average, how many hours/day did you spend doing each of the following things on your trip?

Clinical exposure __________

Leisure time __________

Medical Research/reading __________

Please indicate any other activities you did which took up a large portion of your time. ______________________________________________________________________________________________________________________________________________________________________________________________________________________________________________________________________________________________________________________________________________________________________________________________________

How worthwhile do you feel your sustainability project was?

□ Extremely

□ Considerably

□ Moderately

□ Slightly

□ Minimally

How beneficial do you feel the healthcare you provided to the patients was?

□ Extremely

□ Considerably

□ Moderately

□ Slightly

□ Minimally

Of the reference tools you took with you, list all the ones you actually used on a regular basis.

1. ­­­­_________________________________________
2. _________________________________________
3. _________________________________________
4. _________________________________________
5. _________________________________________

Describe any barriers that prevented you from using the reference tools you took (i.e: lack of Internet, pace of patient care, etc.).

______________________________________________________________________________________________________________________________________________________________________________________________________________________________________________________________________________________________________________________________________________________________________________________________________

Describe how you were received by the local population.

______________________________________________________________________________________________________________________________________________________________________________________________________________________________________________________________________________________________________________________________________________________________________________________________________

Describe what you learned in terms of clinical skills.

______________________________________________________________________________________________________________________________________________________________________________________________________________________________________________________________________________________________________________________________________________________________________________________________________

Describe how this medical relief trip impacted your awareness of global health issues. ______________________________________________________________________________________________________________________________________________________________________________________________________________________________________________________________________________________________________________________________________________________________________________________________________

Did the trip change your outlook on the practice of medicine? How?

______________________________________________________________________________________________________________________________________________________________________________________________________________________________________________________________________________________________________________________________________________________________________________________________________

Did you vote in the most recent presidential election?

□ Yes

□ No

□ Prefer not to answer

Did your awareness of global health issues impact the amount of support you had for the candidate you voted for? Skip this question if not applicable.

□ Yes

□ No

□ Prefer not to answer

Describe some significant differences between how medicine is practiced in the United States and how it is practiced in the country you visited.

______________________________________________________________________________________________________________________________________________________________________________________________________________________________________________________________________________________________________________________________________________________________________________________________________

What changes could have been made to increase your overall level of satisfaction for the trip?

__________________________________________________________________________________________________________________________________________________________________________________________________________________________________________

__________________________________________________________________________________________________________________________________________________________________________________________________________________________________________

Please provide any additional thoughts you had about your overall experience on this medical relief trip.

__________________________________________________________________________________________________________________________________________________________________________________________________________________________________________________________________________________________________________________________________________________________________________________________________________________________________________________________________________________________________________________________________________________________________

Please include any changes we could make to improve the overall quality of this survey.

__________________________________________________________________________________________________________________________________________________________________________________________________________________________________________________________________________________________________________________________________________________________________________________________________________________________________________________________________________________________________________________________________________________________________
